# Focus on Triple-Negative Breast Cancer: Potassium Channel Expression and Clinical Correlates

**DOI:** 10.3389/fphar.2020.00725

**Published:** 2020-05-20

**Authors:** Elena Lastraioli

**Affiliations:** Department of Experimental and Clinical Medicine, University of Florence, Florence, Italy

**Keywords:** potassium channels, breast cancer, triple-negative breast cancer, treatment, prognosis

## Abstract

Despite improvements in early diagnosis and treatment, breast cancer is still a major health problem worldwide. Among breast cancer subtypes, the most challenging and harder to treat is represented by triple-negative molecular subtype. Due to its intrinsic features this subtype cannot be treated neither with hormonal therapy (since it does not express estrogen or progesterone receptors) nor with epidermal growth factor receptor 2 (HER2) inhibitors (as it does not express high levels of this protein). For these reasons, the standard of care for these patients is represented by a combination of surgery, radiation therapy and chemotherapy. In this scenario, searching for novel biomarkers that might help both in diagnosis and therapy is mandatory. In the last years, it was shown that different families of potassium channels are overexpressed in primary breast cancers. The altered ion channel expression may be useful for diagnostic and therapeutic purposes due to some peculiar characteristics of this class of molecules. Ion channels are defined as pore-forming transmembrane proteins regulating passive ion fluxes in the cells. Ion channels represent good potential markers since, being localized at the plasma membrane level, their detection and block with specific drugs and antibodies might be fast and tunable. This review focuses on triple-negative breast cancers and recapitulates the current knowledge about potassium channels' clinical relevance and their potential use in the clinical setting, for triple-negative breast cancer diagnosis and therapy.

## Introduction

Breast cancer (BC) is a heterogeneous disease composed of different molecular subtypes, with peculiar histopathological and biomolecular features. Currently BC classification relies on the detection of four biomarkers: estrogen receptor (ER), progesterone receptors (PgR), human epidermal growth factor receptor 2 (HER2), and the proliferation index (Ki67 staining). Applying such molecular classification, five main BC subtypes with different features and prognosis can be defined: Luminal A, Luminal B, HER2-positive, triple-negative (TNB), and normal-like. Among BC subtypes, the most challenging and harder to treat is represented by triple-negative BC (TNBC) (Santana-Davila and Perez, 2010; Penault-Llorca and Viale, 2012).

In this scenario, searching for novel biomarkers that might help clinicians in BC management appears to be mandatory. Mounting evidences gathered in the last 20 years pointed out that several ion channels might represent biomarkers, since their expression is frequently dysregulated in BC and association with clinico-pathological features have been described for some of them.

The aim of the present review is to summarize the current knowledge about potassium channels relevance in BC with a special focus on their role in treatment and patients' outcome.

## Clinical Features of TNBC

According to data recently published by the American Cancer Society (American Cancer Society, 2017), TNBC accounts for 12% of all BC. Its incidence is higher in young patients, in subjects carriers of BRCA1 mutations (Perou and Børresen-Dale, 2011) and of particular ethnicity (African American and Hispanic women are at higher risk with respect to Caucasians) (Millikan et al., 2008). TNBC patients generally have a bad prognosis since their tumors are often bigger, classified as G3 and with lymphnode involvement (Dent et al., 2007). The main features of TNBCs are summarized in **Table 1**.

**Table 1:** Triple-negative breast cancer (TNBC) characteristics.

CHARACTERISTIC		TNBC	Basal-like BC
**Age of Onset**		<50 years	<50 years
**Ethnicity**		More frequent in African American	More frequent in African American
**Radiological findings**		Hyperdense masses; no calcifications	Hyperdense masses; no calcifications
**Histological features**	Grading Invasive Subtype Growing pattern Lymphocytic infiltrate	G2–G3 Yes Ductal carcinomas of no special type Pushing borders High	G3 Yes Ductal carcinomas of no special type, with Basal-like features Pushing borders High
**Molecular** **features**	ER PgR HER2 CK5 CK17 EGFR Cyclin E	--- --- --- + + + +	- -- - ++ ++ + +
**Prognosis**	After 5 years Relapse (10 years)	intermediate Rare	unfavorable Very rare
**Therapy**	Chemotherapy Trastuzumab Hormonal therapy	Yes (especially combinations with Doxorubicin and taxanes) No No	Yes No No

ER, estrogen receptor; PgR, progesterone receptor; CK, cytokeratin; EGFR, epidermal growth factor receptor; —, negative by definition; –, almost always negative; -, usually negative; +, usually positive; ++, almost always positive.

## Histopathology of TNBC

**Figure 1:**
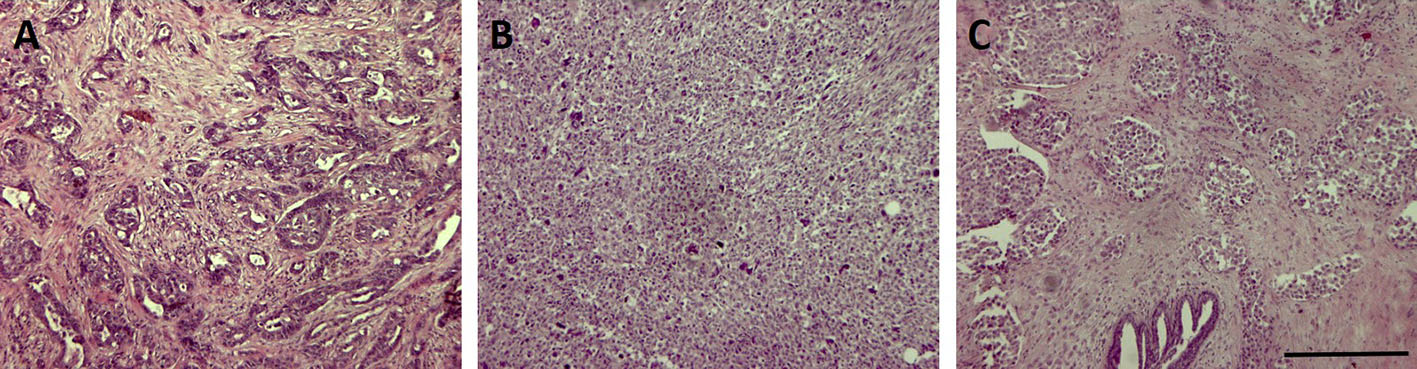
Representative pictures of three triple-negative breast cancer (TNBC) samples of different histotype stained with Hematoxylin & Eosin. Bar: 100µm. **(A)** invasive ductal carcinoma; **(B)** invasive metaplastic carcinoma-spindle cell subtype; **(C)** invasive lobular carcinoma.

From a histopathological point of view, most TNBC are classified as invasive ductal carcinomas of no special type (Carey et al., 2006) that could display basal-like features or not. A minor part of TNBC belongs to rare histological types such as atypical or typical medullary-like (Jacquemier et al., 2005; Livasy et al., 2006), apocrine carcinomas and pleomorphic lobular carcinomas (Kreike et al., 2007), metaplastic carcinomas (Livasy et al., 2006; Reis-Filho et al., 2006), and adenoid cystic carcinomas (Cleator et al., 2007). Among them, it was shown that metaplastic and medullary-like cancers display basal-like features that are uncommon in the other histotypes (Jacquemier et al., 2005; Bertucci et al., 2006; Rodriguez-Pinilla et al., 2007). Three representative pictures of TNBC of different histotype are reported in **Figure 1**.

As summarized in **Table 1**, TNBC are frequently associated with aggressive histopathological characteristics such as an abundant lymphocytic infiltrate and central necrotic area, high grading (either nuclear and histological), high mitotic rate, presence of pushing margins and altered nuclear-cytoplasmatic ratio (Tot, 2000; Fulford et al., 2006; Livasy et al., 2006; Bauer et al., 2007; Kreike et al., 2007; Millikan et al., 2008; Sasa et al., 2008; Kaplan et al., 2009).

## Biomolecular Features of TNBC

Since the terms “TNBC” and “Basal-like” are not synonymous, although they share many common features, a thorough analysis of a great cohort of samples in the METABRIC study according to PAM50 (Curtis et al., 2012; Engebraaten et al., 2013) was performed: such analysis showed that most TNBC are classified as Basal-like (86.1%) but also other subtypes such as HER2-enriched, normal-like and Luminal A are represented.

The gene expression analysis of the same cohort showed that the higher percentage of basal-like tumors do not express ER, PgR and HER2 (and are therefore defined as triple negative), but there are also samples expressing either ER or HER2. Due to the discrepancy between the two classifications, several efforts have been made to provide a more precise classification using immunohistochemical markers such as cytokeratin and EGFR (see also **Table 1**) (Nielsen et al., 2004; Carey et al., 2006; Blows et al., 2010; Broeks et al., 2011).

## TNBC Treatment

As stated before, TNBC represent the most difficult to treat due to the absence of good targets such as hormone receptors or other molecules to be specifically targeted with effective drugs. A recent review (Yu et al., 2020) described some novel potential targets such as gene targets, Noncoding RNA, classical signaling pathways among others. From a clinical point of view, in 2017 the Food and Drug Administration (FDA) approved the use of cyclin-dependent kinases 4/6 (CDK4/6) inhibitors (ribociclib, palbociclib, abemaciclib) for ER+ and HER2- advanced BC (Eggersmann et al., 2019). In the following year, also PARP inhibitors (talazoparib, olaparib) were approved for HER2- and BRCA1/2-mutant advanced BC (McCann and Hurvitz, 2018).

## Potassium Channels

Ion channels have been proven to be expressed in human tumors of different origin and it was shown that they act in different manners, modulating several key cell processes. For these reasons, ion channels could represent novel cancer biomarkers, once properly validated in the clinical setting. Being localized in the plasma membrane, ion channel detection might be easily performed by IHC and molecular techniques thus raising the possibility of quick detection of the protein; moreover, for the same reason, they represent a good potential target for therapy with specific drugs and antibodies.

**Figure 2:**
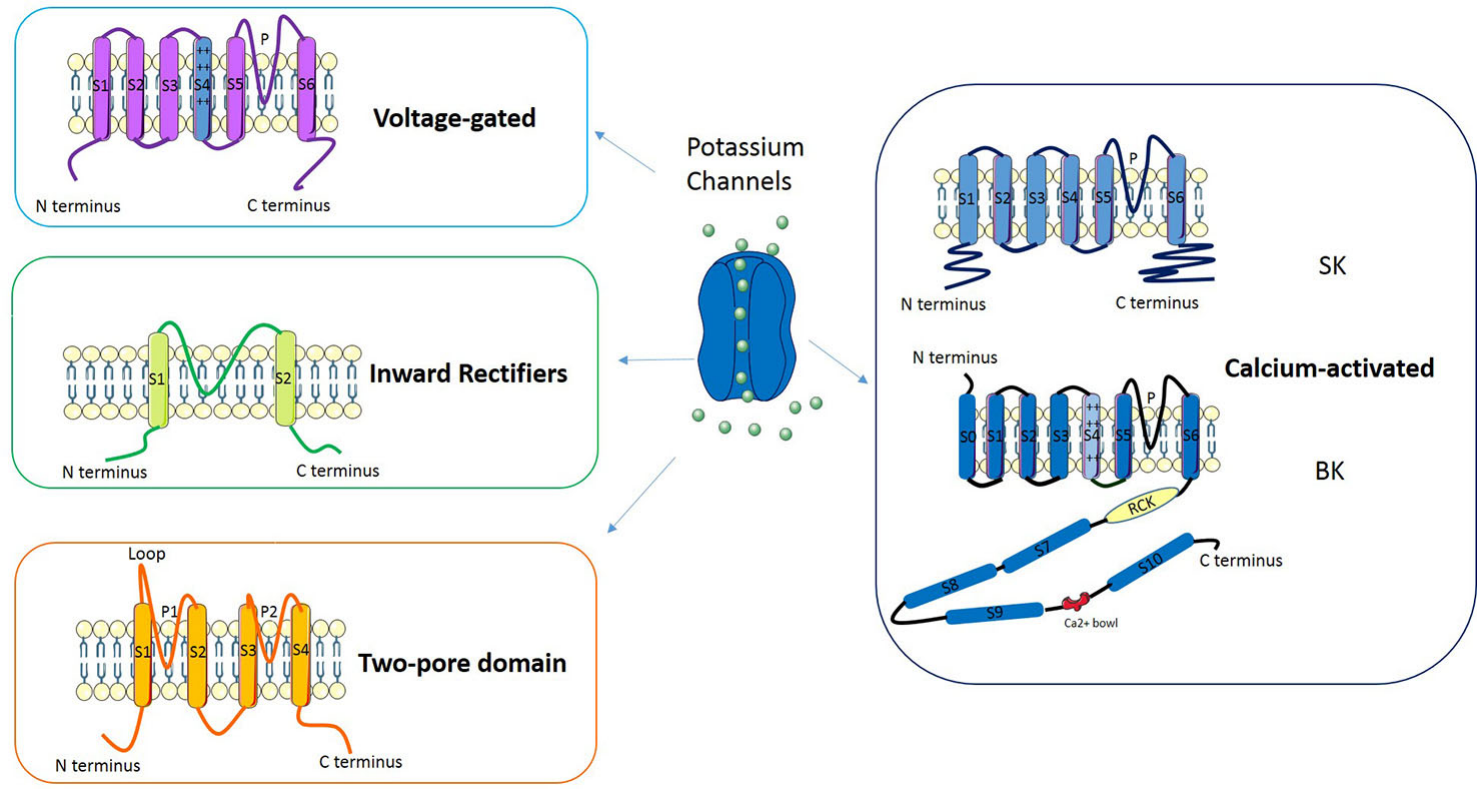
Classification and molecular structure of potassium channels.

Potassium channels are a multi-gene family composed of several subfamilies (**Figure 2**): voltage-gated potassium channels, inward rectifiers, two-pore domains and calcium-activated channels (reviewed in D'Amico et al., 2013). In Voltage-gated potassium channels four subunits surround an aqueous pore: each subunit is composed by six transmembrane domains (S1–S6), of which S4 represents the voltage sensor while the pore (P) is defined by a loop between S5 and S6. The channels belonging to the Inward rectifiers subfamily are composed of four subunits, each with two transmembrane domains linked by a P-loop. Channels classified as Two-pore domains are characterized by four transmembrane domains and an aqueous pore formed by two regions. Another subfamily is represented by Calcium-activated potassium channels: these channels belong to two groups of proteins named “small- and intermediate- conductance” (SK) and “high-conductance” potassium channels (BK). The former are tetramers composed by six transmembrane domains (S1–S6) each with the central pore located in the S5-S6 region; the latter are tetramers made of α and β subunits in which the pore is formed by α subunits.

## Potassium Channels in BC

It is well known that that ion channels are aberrantly expressed in different tumors (see, for example, the review by Lastraioli et al., 2015a). Among them, potassium channels have been shown to be overexpressed in primary BCs and cell lines (reviewed in Lastraioli, 2018). In **Table 2**, a detailed list of the potassium channels whose expression has been described in BC are reported.

**Table 2:** Potassium channels expressed in primary breast cancer (BC).

FAMILY	HGNC name	IUPHAR name	Alternative names	GENE	Clinical correlations
**Voltage-gated**	KCNA3	Kv1.3	MK3, HLK3, HPCN3	*KCNA3*	Poor prognosis (Brevet et al., 2009), advanced stage, younger age (Jang et al., 2009)
	KCNH1	K_v_10.1	eag1	*KCNH1*	Overexpression (Hemmerlein et al., 2006), correlation with vitamin D receptor in invasive ductal carcinomas (García-Becerra et al., 2010), poor prognosis^,^ high expression in TNBC, association with stage, positive lymph nodes, higher expression in invasive ductal carcinomas (Liu et al., 2015)
	KCNH2	K_v_11.1	hERG1	*KCNH2*	*KCNH2* gene overexpression (Fukushiro-Lopes et al., 2017), correlation with molecular subtype, grading, ER, ki67 (Iorio et al., 2018), relapse (Breuer et al., 2019)
**Inward Rectifier**	KCNJ3	Kir3.1	GIRK1, KGA	*KCNJ3*	Lymph node metastases, association with ER, independent prognostic factor (OS and DFS) (Kammerer et al., 2016)
**Two-pore domain**	KCNK5	K2p5.1	TASK-2, TASK2, KCNK5b	*KCNK5*	Overexpression (Dookeran et al., 2017)
	KCNK6	K2p6.1	KCNK8, TOSS, TWIK-2, TWIK2	*KCNK6*	Downexpression (Dookeran et al., 2017)
	KCNK9	K2p9.1	KT3.2, TASK-3, TASK3	*KCNK9*	Gene amplification (Mu et al., 2003; Lee et al., 2012), Overexpression (Dookeran et al., 2017)
	KCNK12	K2p12.1	THIK-2, THIK2	*KCNK12*	Overexpression (Dookeran et al., 2017)
	KCNK15	K2p15.1	KCNK11, KCNK14, KT3.3, TASK-5, TASK5, dJ781B1.1	*KCNK15*	Downexpression (Dookeran et al., 2017)
**Calcium-activated**	KCNMA1	KCa1.1	mSLO1	*KCNMA1*	High stage, high grade, proliferation, poor prognosis (Oeggerli et al., 2012), brain metastases (Khaitan et al., 2009)
	KCNN4	KCa3.1	hSK4, hKCa4, hIKCa1	*KCNN4*	High grade with negative lymph nodes (Haren et al., 2010); poor prognosis (Faouzi et al., 2016)

Kv1.3 channels, are members of the voltage-gated family, encoded by *KCNA3* gene. It has been shown that Kv1.3 channels have a key role in several cell processes such cell proliferation, apoptosis, setting the cell resting membrane potential and regulating cell volume (Cahalan and Chandy, 2009; Teisseyre et al., 2015). Kv1.3 channels have been shown to be associated with poor prognosis in BC patients (Jang et al., 2009). The expression of Kv1.3 mRNA and the corresponding protein was proven to be reduced in grade III BC and an inverse association with tumor grade and advanced stage (Brevet et al., 2009) emerged. The same Authors, demonstrated that the methylation of the promoter of the gene increased in grade III tumors (thus decreasing *KCNA3* transcription) and it is associated with poor differentiation and younger age of the patients (Brevet et al., 2009). Recently, the potential use of Kv1.3 for cancer diagnostic and therapy has been reviewed (Teisseyre et al., 2019).

Kv10.1 (Eag1 or KCNH1) is a voltage-gated potassium channel encoded by the *KCNH1* gene. In physiological conditions, Kv10.1 channels are expressed mainly in brain, adrenal gland, myoblasts, placenta and testis (Bijlenga et al., 1998; Ouadid-Ahidouch et al., 2016). In excitable tissues (muscle and brain) Kv10.1 channel sustains hyperpolarization and controls neuronal excitability (Ouadid-Ahidouch et al., 2016). Nevertheless, Kv10.1 expression has been described also in several human tumors at difference from the corresponding normal tissues both at the mRNA and protein level (Hemmerlein et al., 2006). High Kv10.1 protein levels have been found in human BCs (Hemmerlein et al., 2006; García-Becerra et al., 2010) and it was shown that Kv10.1 expression is higher in invasive-ductal carcinomas than in fibroadenomas (García-Becerra et al., 2010). More interestingly, Kv10.1 is highly expressed in TNBCs with respect to other molecular subtypes and it was shown that it is associated with tumor stage, size, and lymph node involvement (Liu et al., 2015). In BC cell lines it was shown that the combination of Astemizole (Kv10.1 blocker) and gefitinib (EGFR inhibitor) have a synergic effect in impairing proliferation in BC cells expressing both proteins (García-Quiroz et al., 2019a). The same Author also showed that the combined treatment with calcitriol and curcumin or resveratrol had a synergistic effect both *in vitro* and *in vivo* in human mammary tumor cells (García-Quiroz et al., 2019b).

Kv11.1 (also named hERG1) encoded by *KCNH2* gene, is another member of the voltage-gated family that has been shown to be overexpressed in several solid tumors (Lastraioli et al., 2015b) and also in BC (Fukushiro-Lopes et al., 2017; Iorio et al., 2018). Through the analysis of public datasets, it was demonstrated that *KCNH2* gene is overexpressed in BC (Fukushiro-Lopes et al., 2017). We recently showed that Kv11.1 protein expression in primary BCs is associated with molecular subtype (Iorio et al., 2018). In particular, we showed that Kv11.1 scoring was higher in Luminal A (tumors expressing ER, PgR, negative for HER2 expression, with a low proliferation index evaluated through Ki67 expression), progressively decreasing in Luminal B (tumors expressing ER, PgR, positive, or negative for HER2 expression, with a high proliferation index), HER2+ (tumors with high HER2 expression, low or absent ER and PgR and low Ki67) and TNBC tumors (Iorio et al., 2018). Moreover, considering all the molecular subtypes it emerged that patients with high Kv11.1 protein expression have a longer Progression-Free Survival and the same trend was observed for Local Relapse Free-Survival and Metastases-Free Survival (Iorio et al., 2018). A recent meta-analysis focused on *KCNH2* gene expression confirmed our data, showing that high expression of the gene was associated with longer relapse-free survival (Breuer et al., 2019) and more interestingly, the association was restricted to ER-negative patients.

The inward rectifier Kir3.1 (also named KCNJ3) encoded by *KCNJ3* gene, is positively associated with the development of nodal metastases (Stringer et al., 2001). The same channel was proven to be associated with ER expression and its expression was demonstrated to be an independent indicator of poor prognosis in ER-expressing tumors (Kammerer et al., 2016).

Several channels belonging to the two-pore subfamily have been shown to be aberrantly expressed in BCs. The *KCNK9* gene encoding for K_2P_9.1 (also indicated as KCNK9 or TASK3), has been shown to be amplified in BC and the corresponding channel is overexpressed (Mu et al., 2003; Lee et al., 2012). Moreover, KCNK9 mediates cell migration in BC cell lines (Lee et al., 2012).

More recently, a comprehensive analysis was performed in TNBC databases of the TCGA (Dookeran et al., 2017): from such analysis it was demonstrated that in TNBC molecular subtype *KCNK5*, *KCNK9*, and *KCNK12* are overexpressed while *KCNK6* and *KCNK15* are down-expressed. Moreover, alterations in CpG island methylation were significantly associated with TNBC subtype (Dookeran et al., 2017).

In primary BCs the expression of KCa1.1 (also named BK), a member of Calcium-activated subfamily encoded by *KCNMA1* gene, is associated with ER expression (Oeggerli et al., 2012), the development of metastases in the brain (Khaitan et al., 2009), nuclear grade, proliferation, stage and poor prognosis (Khaitan et al., 2009). KCa1.1 was found to be expressed in different BC subtypes and is blocked by Penitrem1 (Goda et al., 2018). The expression of another member of the same family, KCa3.1 (also named KCNN4 and encoded by the *KCNN4* gene) was found to be associated with high grade BCs without lymph nodes involvement (Haren et al., 2010). Another report through the analysis of public datasets highlighted the relevance of KCa3.1 in BC since it was shown that the mRNA levels were associated with poor prognosis (Faouzi et al., 2016).

## Potassium Channels as Therapeutic Targets

Several evidences have been gathered addressing the possibility of using ion channels (and in particular potassium channels) as therapeutic targets (reviewed in Arcangeli et al., 2009). A paper published in 2003 (Abdul et al., 2003) and focused on Kv1.3 channels, demonstrated that potassium channel blockers such as dequalinium and amiodarone significantly reduced MCF-7 BC cells proliferation and increased the growth-inhibitory effects of the anti-estrogen drug tamoxifen on BC cell lines, also those derived from TNBC (MDA-MB231) (Abdul et al., 2003).

In another interesting paper it was shown that Penitrem 1 is capable of blocking BK channels and that Penitrem A has a synergistic antiproliferative effect with HER-targeting drugs (Goda et al., 2018).

A member of the Calcium-activated subfamily, KCa3.1, was found to promote the acquisition of radioresistance in BC cells (Mohr et al., 2019), therefore it was suggested that targeting the channel during radiation therapy could result in radiosensitization of the tumor.

*In vivo*, it was shown that the Kv10.1 blocker astemizole impaired MBCDF and T-47D cells xenografts growth (García-Quiroz et al., 2019a). Moreover, the combined treatment with astemizole and calcitriol caused a significant reduction of the T-47D xenografts tumor masses as it happened in mouse models obtained inoculating primary BCs (García-Quiroz et al., 2014). The molecular bases of such effect were explained in a previous paper of the same group (García-Quiroz et al., 2012): calcitriol has an antiproliferative effect since it inhibits Kv10.1 (that modulates cell cycle and tumor progression) while astemizole is a blocker of Kv10.1 currents; the synergistic effects involve the downregulation of Cytochrome P 24A1 (CYP24A1), upregulation of Vitamin D Receptor (VDR) and Kv10.1 targeting. It was also demonstrated that Chloroquine (a drug used for the treatment of malaria that has been investigated also for its antitumoral effects) inhibited Kv10.1 currents and reduced cell migration in MDA-MB-231 cells (Valdés-Abadía et al., 2019).

Kv11.1 was proven to be a potential therapeutic target in several human solid cancers (Crociani et al., 2013; Crociani et al., 2014; Lastraioli et al., 2015c; Iorio et al., 2020). Long ago, it was demonstrated that Kv11.1 current was inhibited by tamoxifen (Thomas et al., 2003) and more recently it was shown that such inhibition is actually due to the active metabolite of tamoxifen, named endoxifen (Chae et al., 2015).

In a paper published in 2011 it was shown that Arsenic trioxide (a drug used to treat mainly promyelocitic leukemias but also effective on solid tumors) induced apoptosis of MCF-7 BC cells, through Kv11.1 inhibition (Wang et al., 2011). Another paper from the same group confirmed such evidences *in vivo*, in MCF-7 xenografts and also determined that the mechanism underlying tumor growth inhibition was centered on a Kv11.1-mir328 pathway (Wang et al., 2015). Recently it was shown that small molecule activators of the Kv11.1 channel impair tumor growth and metastasis in xenografts of TNBC (Breuer et al., 2019).

More interestingly, the combination of specific channel blockers along with drugs already in use in the clinical settings (such as bevacizumab) was proven to be extremely effective since it was able to significantly reduce *in vivo* tumor growth in mouse models (Crociani et al., 2014). In a recent paper (Iorio et al., 2020), we demonstrated that Kv11.1 (along with HIF2α) has a positive impact on PFS of patients affected by metastatic colorectal cancer and treated with bevacizumab and could therefore be used to identify patients likely to benefit from the treatment. This finding could be of particular interest also in BC treatment, since the approval to use bevacizumab for metastatic BC patients was revoked by the FDA in 2011 due to the little improvement in favorable outcome considered not sufficient. Nevertheless, a meta-analysis of randomized controlled trials showed that the combination of chemotherapy and bevacizumab significantly improved PFS of metastatic BC patients and could therefore be used, once selected the optimal target population (Li et al., 2015).

In a different setting (pancreatic ductal adenocarcinoma), we found an association and a physical interaction between Kv11.1 and EGFR, raising the possibility of designing combined treatment schedules, also involving Kv11.1 blockers (Lastraioli et al., 2015c). Also in this case, such therapeutic approach could be applied to BC patients.

## Conclusions

TNBC is characterized by a poorer prognosis with respect to other BC subtypes. Due to the absence of molecular markers there is currently no targeted therapy and the gold standard of therapy for TNBC is represented by chemotherapy. For these reasons, searching for novel biomarkers and targets for TNBC is mandatory. In this scenario, ion channels have a great potential of representing potential biomarkers due to their localization that allows an easy detection for diagnostic and prognostic purposes and could also be easily blocked by specific drugs and antibodies.

## Author Contributions

EL contributed solely to the article.

## Conflict of Interest

The author declares that the research was conducted in the absence of any commercial or financial relationships that could be construed as a potential conflict of interest.
